# Is There Any Room for Women in Medical Research? Bioethical Concerns

**DOI:** 10.4274/balkanmedj.galenos.2020.2020.2.001

**Published:** 2020-02-28

**Authors:** Berna Arda

**Affiliations:** 1 Department of History of Medicine and Ethics, Ankara University Faculty of Medicine, Ankara, Turkey

Have you ever seen a female figure in Rembrand’s famous Dr. Tulp’s anatomy lesson? Neither the professor who teaches, nor the surroundings, nor the cadaver is a woman. This painting from 1632 is, in fact, quite compatible with reflecting the realities of traditional medicine, successfully representing a medical environment in which women have not found a place for a long time. Throughout history, the doctor has been described as a male one in the physician oaths, who learns the profession from his father, and this training was transferred to sons, not to daughters. So, throughout ages, medicine has been, in practice, formally was closed to female physicians. During the medieval times in the western world, women healers were under great threat, as they were regarded as witches by the inquisition and severely punished. In fact, they were practitioners of folk medicine and, also, the guardians and developers of empirical medical knowledge based on the huge experience from their predecessors. Undoubtedly, as a result of a difficult and challenging process, women have joined the official practitioners of medicine roughly around the 19^th^ century (1).

Is gender equity only a topic of basic human rights? Or does it represent just a matter from a justice point of view? Is there any meaning of it from scientific practice and research in medicine? It seems like one of the most crucial points to produce the best research and provide the best care to patients. At first, we have to recall the basic definitions of sex and gender, and this would let us find the reflections of gender issues in medicine.

“Sex” describes some characteristics brought by birth based on the reproductive functions of people and emphasizes the biological dimension; on the other hand, “gender” looks a close concept but actually is far beyond this dimension. It has been pointing to a concept that is shaped by social and cultural factors. In the light of gender concept, we have to be aware that these are the roles of femininity and masculinity that are not brought by birth but instead have been determined socially and culturally. There are rigid prejudgements that show differences between societies. Especially, when the references that are made to social and cultural differences are considered, the gender term is also used wider to denote a range of identities that do not correspond to established ideas of men and women.

According to the relevant literature medicine, the ideology of gender roles, gender blindness, gender inequality, and masculine bias do strike us as the realities for medicine today. It is common for men to be in a risky behavior pattern resulting in disability and even death, to deny their illnesses, to disrupt their control, and to not get their treatments properly. Therefore, sexually transmitted diseases such as human immunodeficiency virus, traffic accidents, and suicides have increased men’s health-threatening risks (2).

The normalization of the masculine attitude is one of the results of the sexist approach in diagnosis and treatment. For example, boys are diagnosed with hyperactivity disorder and behavior disorder more than girls. The acceptance of boys’ behaviors being correlated with masculinity causes treatment to begin late (3).

Medicine and gender blindness, power asymmetry, and gender norms between men and women lead to bias. The fact is that the researches are mostly based on men and that the male body alone leads to the overlooking of gender, environment, and psychosocial effects in treatment too. It is a historical fact that medical education is structured almost exclusively on male anatomy.

According to the relevant literature on “gender blindness,” male health workers are more gender blind than women. In a study including family physicians on violence against women in the Netherlands, some of the male doctors have stated that refusing to have sex can provoke violence and that all female doctors involved in the study have stressed that there is no justification for using sexual violence (4).

Gender expresses the set of roles, behaviors, and values that are created by dominancy of masculine thinking in society, attributed to gender by society, and lead to continuing a sort of disadvantageous model of inequality for women. There are striking examples of masculine thoughts and behaviors that leads to bias. For example, the data regarding the coronary artery diseases associated with men were more than the data of rheumatic diseases considered specific to women (5). Therefore, while coronary artery diseases can be prevented by physical activity-nutrition recommendations in the early period, women may be deprived of protective measures because of the biased attitude that does not pay regard to risk for women. Another example is in the field of psychiatry. On the one hand, the frequency of depression is twice as high in women as in men. How much of this difference stems from the fact that men do not apply to hospitals because of their gender roles? Or what is the effect of not being diagnosed with depression in men presenting? Do we know this exactly? On the other hand, the frequency of suicide in men is higher than in women (6). What would be the consequences of the lack of a gender-sensitive approach to diagnosing depression?

Bioethics as an academic field is interested in value problems to identify and to analyze with a critical approach. Therefore, the problems that depend on gender are natural subjects of bioethics. The author’s training experience on gender and bioethics may seem to be limited despite teaching for more than 20 years in Ankara University Gender Studies Master Program since 1997, and this might be considered as one of the rare samples in the field. One of the taught courses in the postgraduate program has been “Bioethics and Woman.” Being one of the 14 courses in the master’s program, the second-year course in the spring term is a mandatory one. It is taught 3 h in a week and has a total of 45 h in one semester. There are no prerequisites to enroll. Interactive seminars, small group discussions, and structured interviews have been conducted. Participation has a transdisciplinary character, and the program is open to students with different backgrounds such as philosophy, psychology, public administration, education, social services, law, archeology, journalism, and medicine (7). There are many situations in which women were in the center and require elaborate bioethical evaluation. In this context, it is the fact that surrogacy has become almost completely commercial motherhood today as a big part of a huge industry. Another fact that infertility is primarily fictionalized over the woman in terms of both diagnosis and treatment is among the bioethical topics that raise concerns about women.

When we look at medical research and women’s perspective, how far are we today from the description of Rembrandt? How much of the research is done for women? How many of the researchers are women? How many of the Nobel Awards in Science, as a criterion, were given to women? We will answer these questions with small numbers and low rates. The world of research still continues to be an environment where women are considered as a minority in every sense.

We have a long way to go to be able to tackle discrimination in scientific research and publishing. In this context, there is a great need for taking modest steps as the Balkan Medical Journal does (8,9), according to SAGER directives (10).
